# Physical distancing versus testing with self-isolation for controlling an emerging epidemic

**DOI:** 10.1038/s41598-023-35083-x

**Published:** 2023-05-20

**Authors:** Stephen C. Newbold, Madison Ashworth, David Finnoff, Jason F. Shogren, Linda Thunström

**Affiliations:** 1 Department of Economics, University of Wyoming, Laramie, WY 82071 USA; 2 Fletcher Group, Inc., London, KY 40741 USA

**Keywords:** Infectious diseases, Health care economics

## Abstract

Two distinct strategies for controlling an emerging epidemic are physical distancing and regular testing with self-isolation. These strategies are especially important before effective vaccines or treatments become widely available. The testing strategy has been promoted frequently but used less often than physical distancing to mitigate COVID-19. We compared the performance of these strategies in an integrated epidemiological and economic model that includes a simple representation of transmission by “superspreading,” wherein a relatively small fraction of infected individuals cause a large share of infections. We examined the economic benefits of distancing and testing over a wide range of conditions, including variations in the transmissibility and lethality of the disease meant to encompass the most prominent variants of COVID-19 encountered so far. In a head-to-head comparison using our primary parameter values, both with and without superspreading and a declining marginal value of mortality risk reductions, an optimized testing strategy outperformed an optimized distancing strategy. In a Monte Carlo uncertainty analysis, an optimized policy that combined the two strategies performed better than either one alone in more than 25% of random parameter draws. Insofar as diagnostic tests are sensitive to viral loads, and individuals with high viral loads are more likely to contribute to superspreading events, superspreading enhances the relative performance of testing over distancing in our model. Both strategies performed best at moderate levels of transmissibility, somewhat lower than the transmissibility of the ancestral strain of SARS-CoV-2.

## Introduction

The United States initially attempted to combat the spread of SARS-CoV-2, the virus that causes COVID-19, using a portfolio of controls that is heavy on physical distancing and masks and light on regular diagnostic testing with self-isolation^1,2^. The widespread use of physical distancing measures—which we construe broadly to include work-from-home requirements, complete or partial school and business closures, inter- and intra-national travel restrictions, and related mandates or recommendations for voluntary behavioral changes that will limit inter-personal contacts—were particularly important in the early stages of the pandemic, before reliable diagnostic tests and effective vaccines and treatments for COVID-19 were widely available. However, these same measures also have led to reduced employment, lost earnings, and a variety of adverse physical and mental health impacts due to withdrawing from economic activities and curtailing social interactions for long periods^3–5^.

A number of prominent researchers and public health experts have advocated widespread and frequent diagnostic testing for mitigating the COVID-19 pandemic^6–8^. These pleas are supported by numerous epidemiological and economic modeling studies, which on the whole suggest well-implemented programs of testing with self-isolation can substantially improve upon a policy approach that relies predominantly on physical distancing^9–15^. None of these studies, however, accounted for the heterogeneity in transmissibility of infected individuals, where a large share of secondary transmissions are caused by a relatively small share of infected individuals^16,17^. Such “superspreading” transmission is a common feature of infectious diseases^18^. For example, Woolhouse et al. found that in 9 of 10 datasets, 20% of infected individuals were responsible for at least 80% percent of secondary infections^19^, and in a review of the role of superspreading in infectious disease outbreaks, Stein suggested that models excluding this feature “will inaccurately portray pathogen dynamics and hinder the successful implementation of infection control strategies^20^”.

We developed an integrated epidemiological and economic model (an ‘epi-econ model’) by coupling a compartment model of an infectious disease outbreak with an economic model of the benefits and costs of the associated health outcomes and control measures. We used our epi-econ model to compare two distinct control strategies—physical distancing versus testing with self-isolation—using a common set of assumptions and parameter values. The model includes the possibility of transmission by superspreading to allow a comparison of control strategies when a disproportionate share of secondary transmissions are caused by a small fraction of infected individuals, as seems to be indicated for COVID-19^17,21–23^.

We also examined the performance of the testing strategy using relatively inaccurate diagnostic tests, since such rapid tests may be the most cost-effective basis for a widespread and frequent random testing program. Specifically, we examined the influence of false negative and false positive testing error rates, the time delay between administering a diagnostic test and reporting the result, and the self-isolation compliance rate on the performance of an optimized testing program relative to optimized physical distancing measures. We also decomposed the costs of each strategy into lost economic productivity or earnings from physical distancing or isolation, which will be borne mainly by households and firms, and the cost of diagnostic testing, a large share of which may need to be subsidized by local or state governments to achieve sufficiently high rates of testing.

We designed this study to provide insights about the relative performance of physical distancing and testing with self-isolation control strategies not only for COVID-19 but also for other pathogens that fall within the broad parameter space considered here. Our primary results are based on parameter settings meant to represent the ancestral strain of SARS-CoV-2, which circulated around the world between January 2020 and the final months of 2021. For comparison, we also present results from alternative parameterizations meant to encompass a wide range of possible variants—including Omicron, which is more transmissible but apparently less lethal than prior SARS-CoV-2 variants—as well as additional sensitivity analyses. Therefore, the results from this study should be useful for informing our response to possible future waves of COVID-19 fueled by other more or less transmissible or lethal variants, or an entirely different contagious respiratory pathogen that might emerge in the future^24^.

An important set of parameters in our model includes those characterizing the accuracy of the diagnostic tests that would support a control strategy based on regular testing with self-isolation, and the rate of compliance with recommendations by public health authorities to isolate upon receiving a positive test result. Diagnostic tests for COVID-19 are known to be imperfect^25,26^. They fail to identify some individuals who are in fact infected (false negatives) and they erroneously identify as infected some individuals who are not (false positives). Furthermore, some people who receive a positive test result may choose not to comply with guidance to isolate for a variety of reasons. They may have work, family, or personal obligations that are overriding, or they may be “conscientious objectors” who do not believe the virus poses a severe enough threat to justify restrictions on individuals’ freedom of movement and association even for those who are infected^27^. Among our main goals in this study is to examine how robust a random testing and self-isolation policy is to these limitations. What are the highest levels of testing error rates and non-compliance that a regular testing and self-isolation policy could exhibit and still provide positive net benefits or perform better than an optimized physical distancing policy? Some of the key elements of our model were chosen to accommodate these important features of a testing program.

A brief description of the key elements of our model follows, and a complete description can be found in the Methods section. Our point of departure is a continuous-time compartment model based on a standard *S-I-R* framework^28^. We added a primary compartment for infected individuals with elevated transmissibility (“superspreaders”)^18^ plus 13 secondary compartments to track the fate of individuals who get tested for COVID-19, including individuals who are waiting for a test result and those in self-isolation. The model includes parameters representing the share of infected individuals who become superspreaders (*k*), the delay in receiving a diagnostic test result ($\sigma$), the false positive and false negative error rates of the tests ($\varepsilon _1$ and $\varepsilon _2$), the average compliance rate among individuals who receive a positive test result and are asked to self-isolate ($\lambda$), the reduced frequency of inter-personal contacts due to physical distancing measures (*x*), and the frequency of random diagnostic testing ($\tau$). The regulator’s task is to choose the level (*x*) and duration ($T_x$) of physical distancing, or the frequency ($\tau$) and duration ($T_{\tau }$) of random testing, or both, to maximize the net benefits of the control measures, which comprise the value of reduced mortality risks minus the value of lost economic output both during the period of implementation and during the period of economic recovery. We compute the value of mortality risk reductions using a central estimate of the “value per statistical life” (VSL)^29^, and we use a concave utility function whose curvature is controlled by the coefficient of relative risk aversion ($\eta$), which implies a downward sloping demand curve for mortality risk reductions^30^.

## Results

**Table 1:** Optimal distancing, testing, and combined policies under four model variations.

	*x*	$T_x$	$\tau$	$T_{\tau }$	Deaths	Value of reduced mortality	Short run output loss	Short run cost of testing	Long run loss	Net benefits
With superspreading, with diminishing VSL ($k=0.1$,$\eta =1$)										
No controls	0.000	0	0.000	0	0.00894	0.000	0.000	0.000	0.000	0.000
Distancing	0.189	141	0.000	0	0.00687	0.249	0.057	0.000	0.091	0.102
Testing	0.000	0	0.522	304	0.00316	0.550	0.061	0.057	0.185	0.247
Combined	0.000	0	0.522	304	0.00316	0.550	0.061	0.057	0.185	0.247
Without superspreading, with diminishing VSL ($k=0$,$\eta =1$)										
No controls	0.000	0	0.000	0	0.00894	0.000	0.000	0.000	0.000	0.000
Distancing	0.189	141	0.000	0	0.00687	0.249	0.057	0.000	0.091	0.102
Testing	0.000	0	0.399	224	0.00509	0.413	0.043	0.034	0.121	0.216
Combined	0.000	0	0.399	224	0.00509	0.413	0.043	0.034	0.121	0.216
With superspreading, with constant VSL ($k=0.1$,$\eta =0$)										
No controls	0.000	0	0.000	0	0.00894	0.000	0.000	0.000	0.000	0.000
Distancing	0.386	334	0.000	0	0.00023	1.207	0.294	0.000	0.461	0.452
Testing	0.000	0	0.995	327	0.00031	1.196	0.085	0.098	0.287	0.727
Combined	0.096	121	0.753	342	0.00018	1.214	0.102	0.084	0.291	0.737
Without superspreading, with constant VSL ($k=0.1$,$\eta =0$)										
No controls	0.000	0	0.000	0	0.00894	0.000	0.000	0.000	0.000	0.000
Distancing	0.386	334	0.000	0	0.00023	1.207	0.294	0.000	0.461	0.452
Testing	0.000	0	1.000	338	0.00092	1.112	0.090	0.101	0.299	0.622
Combined	0.139	198	0.690	343	0.00018	1.215	0.131	0.080	0.330	0.674

Deaths are reported per capita, and capitalized monetary values are reported as a fraction of annual GDP.

Our main results from four model variations, which are distinguished by whether or not we include superspreaders or a diminishing VSL, are presented in Table 1. The first row in each section of the table represents the uncontrolled scenario, under which 0.894% of the population dies from infection. No benefits or costs accrue under this scenario because no policy controls are implemented.

Under the first model variation, which includes superspreading and a diminishing VSL, the optimal distancing policy reduces the contact rate by $[1-(1-0.189)^2] \times 100 = 34.2$% of its uncontrolled level for around four and a half months, which reduces deaths from infection by 23%. The present value of benefits of this policy are equivalent to 24.9% of annual GDP, and the total (short- plus long-run) costs are 14.8% of GDP, so the net economic benefit is equal to 10.2% of GDP. Under the optimal testing policy, everyone who is not already isolated or waiting for a test result is tested about every other day (52.2% of days) for about 10 months. This policy reduces deaths from infection by 67.4%, with benefits, costs, and net benefits of 55.0%, 30.3%, and 24.7% of GDP. Under this model variation, we could find no combination of distancing and testing that could improve on the optimal testing strategy.

Under the second model variation, we removed superspreaders from the system by setting $k\!=\!0$. We then re-solved the distancing, testing, and combined policy optimization problems to isolate the influence of superspreaders by contrast to the outcomes under the first model variation. The qualitative results are similar to the case with superspreaders: the testing strategy performs better than the distancing strategy, and again we found no combined strategy that could improve upon the optimal testing strategy. Two notable results are that the performance of the optimized distancing policy is the same with and without superspreaders, and the performance of the testing policy is lower without superspreaders than with. The reason for the former result is that the *S-I-R* model is calibrated to the same $R_0$ value with or without superspreaders, and we assumed that the behaviors of infected and superspreading individuals are the same. What distinguishes them is a higher viral load^31^, not a higher rate of social mixing by superspreaders. While our model explicitly incorporates only the first factor, in reality superspreading may stem from either factor or both factors working together^32^. This assumption also explains why the optimized testing policy performs better with superspreaders. We assumed that the sensitivity of diagnostic tests is positively related to the viral load of the test subjects, so superspreaders are more likely to be identified by testing than are non-superspreading infected individuals^33^. In some cases, rapid tests that fail to detect individuals with low viral loads may be preferred to very sensitive tests. Such low-sensitivity tests can catch superspreaders but would allow individuals with lower viral loads who have a much lower risk of spreading the virus to pass through the detection net, thereby saving the cost of unnecessary isolation. Also note that because we calibrated *k* and $\alpha$ such that $R_0$ remains fixed, this influence of superspreading is separate and apart from any differences in the performance of the two policy strategies at different levels of $R_0$.

Under the third model variation, we included superspreaders again by setting $k = 0.1$, but we made the VSL constant rather than diminishing with the size of the risk reduction by setting $\eta = 0$. In this variation, both optimized policies are more stringent than under the second model variation because the benefits of control are higher with a constant VSL, all else equal. As before, the optimized testing strategy significantly outperforms the optimized distancing strategy. In this case, the combined policy performs slightly better than the optimized testing policy alone. Adding a small amount of distancing ($x=0.096$) for around three months ($T_x = 121$ days) and somewhat relaxing the rate and duration of testing increases the net benefits slightly, from 72.7 to 73.7% of GDP.

Under the fourth model variation, we excluded superspreading and used a constant VSL. The optimal distancing policy is the same as that under the third model variation for the same reasons that the optimal distancing policy did not differ between the first two model variations, as explained above. In this case the optimal testing policy is extended 11 additional days, but the policy is less effective than under the third model variation because the relative advantage testing has in identifying individuals with higher than average viral loads has been lost without the superspreaders. As in the third model variation, the combined policy slightly outperforms the optimal testing policy by adding a modest amount of distancing for nearly seven months. A final note on the results shown in Table 1 is that in each of the four scenarios the most economically efficient policy (with the maximum net benefits) also is the policy with the fewest mortalities.

**Figure 1:**
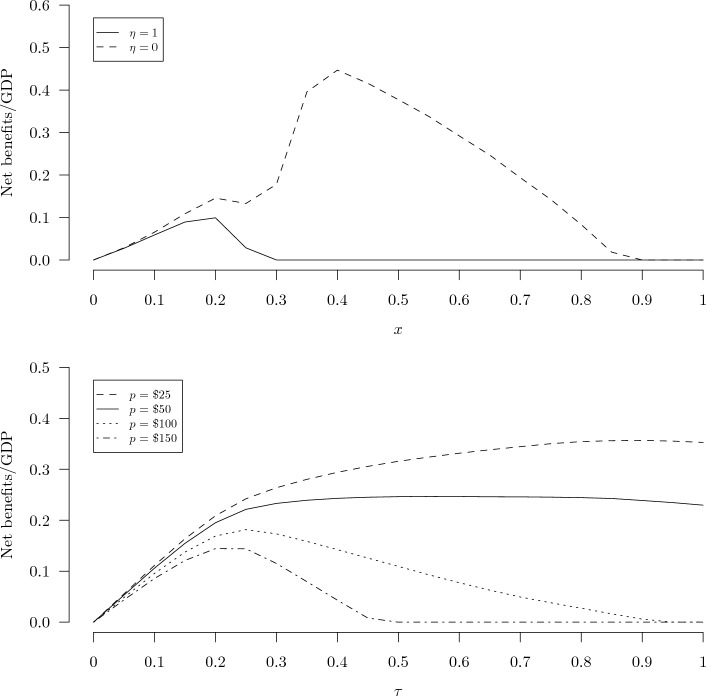
Net benefits (as a fraction of GDP, *y*-axis) of the physical distancing policy (top) and the random testing and self-isolation policy (bottom).

We performed a series of sensitivity analyses to better illustrate the behavior of the model and to examine the influence of several key parameters. Figure 1 shows the net benefits of the physical distancing policy over the full range of the distancing fraction, *x*, from 0 to 1 at two levels of the coefficient of relative risk aversion, $$\eta$$ = 1 and 0 (top panel), and the testing policy over a range of testing rates, $$\tau$$, from 0 to 1 per day at four different assumed prices of the tests, *p* = $25, $50, $100, and $150 (bottom panel). In each case the duration of the policy—$$T_x$$ for the distancing policy and $$T_{\tau }$$ for the testing policy—has been optimized conditional on *x* or $$\tau$$. With $$\eta = 1$$, the net economic benefit of the physical distancing policy reaches a global maximum at around $$x=0.2$$. With $$\eta = 0$$, the net economic benefit of the physical distancing policy initially increases to a local maximum around $$x=0.2$$, then declines slightly, then increases again to its global maximum around $$x = 0.4$$, then decreases smoothly to zero around $$x=0.9$$. The curves do not drop below zero because $$T_x$$ is optimized simultaneously, so if *x* is too high then $$T_x$$ will be reduced to zero if necessary. The bottom panel shows that, at our primary test price *p* = $50, the net economic benefit of the random testing policy increases relatively rapidly from $$\tau = 0$$ to about 0.25 then remains relatively flat but maximum value around $$\tau =0.5$$. The additional curves for *p* = $25, $100, and $150 show that the optimal testing rate decreases from close to 1 at low test prices down to around 0.2 for sufficiently high test prices.

**Figure 2:**
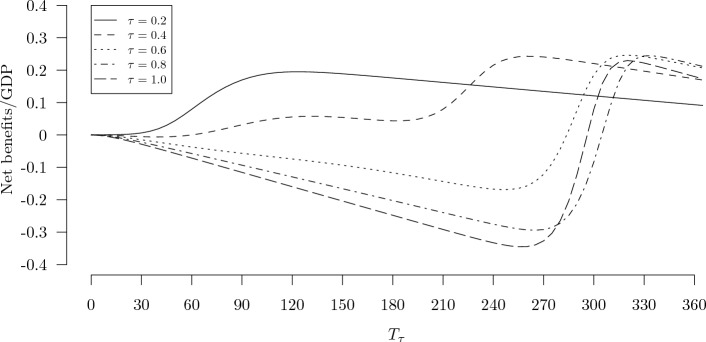
Net benefits (as a fraction of GDP, *y*-axis) of the random testing with self-isolation policy for $\tau$ = 0.2, 0.4, 0.6, 0.8, and 1.0 over the range of $T_{\tau }$ from 0 to $T_{v} = 365$ (*x*-axis).

To help explain the behavior of the curves shown in Fig. 1, net benefit curves across the full range of the testing policy duration, $T_{\tau }$—each one holding the testing rate constant at $\tau =$ 0.2, 0.4, 0.6, 0.8, or 1.0—are shown in Fig. 2. The solid curve shows that the optimal duration of the testing program for $\tau = 0.2$ is around 120 days, and there is only one local maximum in this case. The dashed and dotted curves show that there can be two local maxima when $\tau$ is between 0.2 and 0.6: for $\tau = 0.4$, one local maximum occurs around 120 days and the other around 250 days. The latter maximum is the global optimum for this testing rate. The remaining curves show that the local maximum at the lower values of $T_{\tau }$ disappears by the time $\tau$ has reached 0.6. The shapes of these curves are due to the fact that if the testing rate is high, then as long as the testing program is in place viral transmission will be suppressed. If the program is ended prematurely, then the epidemic can re-ignite and cause a second wave of infections that peaks before the vaccine arrives. For example, the policy with $\tau = 1.0$ and $T_{\tau } = 250$ days would lead to a large net *loss*. In this case, a high cost of testing will accrue over the course of the testing program, but after testing ceases enough time remains for nearly the full curve of infections to peak and decline before the vaccine arrives. Many resources will have been wasted just to delay nearly the same sized peak until later in the year with nearly the same number of deaths as in an uncontrolled scenario. This highlights the important role of the anticipated vaccine arrival time, $T_v$.

**Figure 3:**
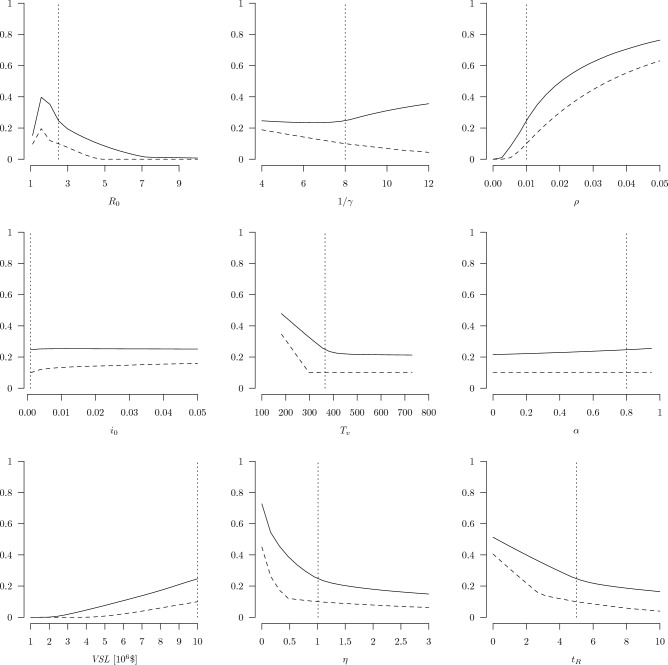
Sensitivity of net benefits (as a fraction of GDP, *y*-axes) of optimal testing and self-isolation policy (solid lines) and optimal physical distancing policy (dashed lines) with respect to $R_0$, $1/\gamma$, $\rho$, $i_0$, $T_v$, $\alpha$, *VSL*, $\eta$, and $t_R$ (*x*-axes). Vertical dotted lines indicate primary values of each parameter, which together produce the main results shown in Table 1.

**Figure 4:**
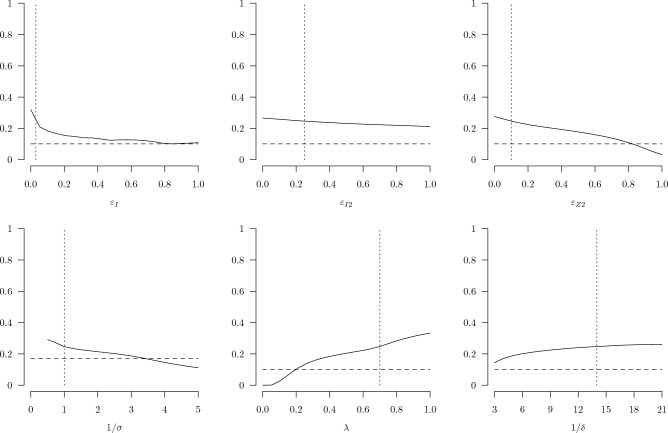
Sensitivity of net benefits (as a fraction of GDP, *y*-axes) of optimal testing and self-isolation policy with respect to: $\varepsilon _1$, $\varepsilon _{I2}$, $\varepsilon _{Z2}$, $1/\sigma$, $\lambda$, and $1/\delta$ (*x*-axes). The solid lines depict the optimized net benefits of the testing policy, the vertical dotted lines correspond to our primary values for each parameter (shown in Table 2), and the horizontal dashed lines correspond to the optimized net benefits of the physical distancing policy.

Results from sensitivity analyses that compare the performance of physical distancing and random testing under a range of assumed values for parameters that are common to both strategies are shown in Fig. 3. Key results illustrated in the panels from left to right and top to bottom are as follows. Holding all other parameters at their primary values, testing outperforms distancing over the full range of $$R_0$$ values. Beyond $$R_0 \approx 5$$, no configuration of the distancing policy has positive net benefits, while some configurations of the testing strategy can be beneficial for $$R_0$$ values at least up to 7. Both strategies perform best when $$R_0$$ is slightly less than 2. The net benefit of distancing declines while the net benefit of testing increases with the average infectious period, $$1/\gamma$$. The net benefits of both strategies increase with the infection fatality rate, $$\rho$$. A notable distinction between the two strategies along this dimension is that the performance curve for the testing strategy separates from the *x*-axis at a lower value of $$\rho$$: even for very low levels of lethality, some configuration of the testing strategy can be beneficial while the distancing strategy cannot. The performance of both strategies changes little with the initial fraction of the population that is infected, $$i_0$$, so both strategies appear to be reasonably robust to their start dates. The performance of both strategies declines as the anticipated arrival time of the vaccine increases. The testing strategy outperforms the distancing strategy at all levels of $$T_v$$ considered, between one half and 2 years. The performance of both strategies declines linearly with $$T_v$$ up to around 300 days for the distancing strategy and nearly 400 days for the testing strategy. After those points, both strategies maintain their performance because herd immunity has been reached by this time. The performance of the testing strategy increases slightly with the share of infections attributable to superspreaders, $$\alpha$$, while the distancing strategy is not affected by this parameter. Testing is beneficial for VSL values greater than about $2 million, while distancing is beneficial for values above around $4 million. The net benefit of both strategies declines with $$\eta$$, rapidly for values of $$\eta$$ below 1, then far less rapidly for values between 1 and 3. The testing strategy still outperforms the distancing strategy at all levels of $$\eta$$ between 0 and 3. The net benefit of both strategies also declines with $$t_R$$, the time required for the output gap to shrink by 95%. As for all other parameters shown in the figure, the testing strategy outperforms the distancing strategy over the full range of $$t_R$$ considered.

**Figure 5:**
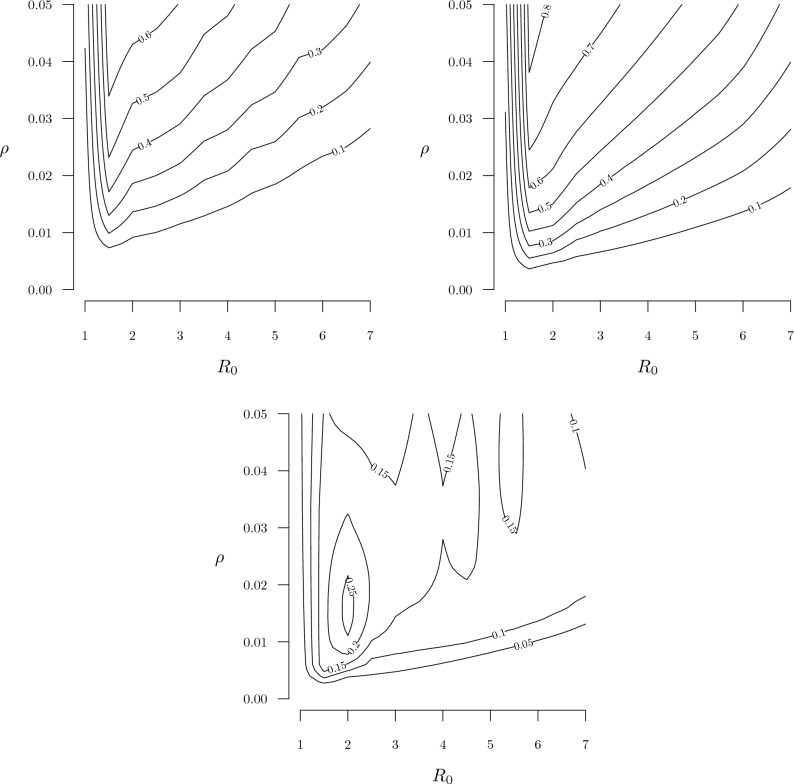
Contour plots of the net benefits of physical distancing (top left), random testing with self-isolation (top right), and the difference between the two (bottom) over a wide range of pathogen transmissibility ($R_0$) and disease lethality ($\rho$).

Results from sensitivity analyses that compared the performance of the optimized testing with that of the distancing policy under a range of assumed values for parameters unique to the testing policy are shown in Fig. 4. Net benefits of random testing remain larger than that for physical distancing over most of the ranges of each parameter in turn—with a few notable exceptions—holding all other parameters fixed at their primary values. The performance of an optimized testing strategy is maximized at a type I (false positive) error rate of 0, and only at very high type I error rates does the performance of testing decline to essentially match the performance of the optimized physical distancing strategy. At this point the testing strategy erroneously catches nearly all those who are tested and the false positives are taken out of circulation, which functions like physical distancing. The performance of the testing strategy declines slowly with the type II (false negative) error rate for non-superspreading infected individuals. The performance of the testing strategy declines more rapidly with the type II error rate for superspreading individuals, and at sufficiently high levels of $\varepsilon _{Z2}$ the testing strategy performs worse than distancing. The performance of the testing strategy declines with the average wait time for test results, as expected, but still outperforms the optimized distancing policy at wait times up to nearly 3.5 days. The testing strategy outperforms the optimized distancing policy for all levels of compliance greater than around 20%. Even at such low compliance, the bias towards identifying superspreaders and the distancing-like effect of isolating false positives appears to make the testing strategy relatively robust to non-compliance with the self-isolation guidelines. The performance of the testing strategy increases slowly with the average duration of self isolation. Beyond an isolation period of around 15 days, the benefits of lengthening the duration of the isolation period are nearly offset by the costs, so the performance curve nearly flattens. The ranges of testing parameters examined in Fig. 4 are meant to safely cover the plausible values for these parameters, so overall these results suggest that a testing policy can perform at least as well as a physical distancing policy throughout a large volume of the plausible parameter space.

Next, we examined the performance of the two strategies over a wide range of combinations of transmissibility ($R_0$) and lethality ($\rho$). The results of these sensitivity analyses are shown as contour plots in Fig. 5. The top left plot shows net benefit contours for the distancing policy, and the top right plot for the testing policy. Both sets of contours exhibit a negative marginal rate of substitution between transmissibility and lethality when $R_0$ is less than around 1.5 (the contour lines slope down), but the MRS is positive for higher levels of transmissibility (the contour lines slope up). In both cases, the best performance occurs when $R_0$ is around 1.5–2.0 and $\rho$ is high. The third plot shows contours of the the difference between the testing and distancing net benefits. No 0-contour appears on the plot, which indicates that the testing policy outperforms the distancing policy over the entire range considered here, and the performance gap is maximized when $R_0 \approx 2$ and $\rho \approx 0.015$.

**Table 2:** Monte Carlo uncertainty analysis results.

	Optimal	No controls	Distancing	Testing
No controls	0.044 (0.009)	–	0.252 (0.019)	0.046 (0.009)
Distancing	0.170 (0.017)	0.748 (0.019)	–	0.360 (0.021)
Testing	0.516 (0.022)	0.954 (0.009)	0.596 (0.022)	–
Combined	0.270 (0.020)	0.956 (0.009)	0.786 (0.018)	0.440(0.022)

Frequency that each policy strategy (No controls, Distancing, Testing, Combined) was optimal among 500 Monte Carlo iterations (column 1), and frequencies that the strategy in each row outperformed each other strategy (columns 2–4). Parameters were drawn from independent triangular distributions with modes and endpoints defined by primary values and ranges shown in Table 3. Numbers in parentheses are standard errors.

Finally, we conducted a Monte Carlo uncertainty analysis by placing triangular distributions on all model parameters with modes equal to the primary estimates and lower and upper bounds corresponding to the ranges for each parameter shown in Table 3. We drew random sets of parameters from these uncorrelated distributions and re-solved the distancing, testing, and combined policy optimization problems under each set of parameters. The frequency of Monte Carlo iterations in which each strategy was optimal and the frequencies that each strategy outperformed each other strategy are shown in Table 2. Distancing alone was optimal in 17.0% of cases, testing alone was optimal in 51.6% of cases, and the combined strategy outperformed both sole strategies in 27.0% of cases. The improvement in net benefits of the combined strategy over testing alone was modest but not negligible: among those random parameter draws where testing outperformed distancing and the combined policy outperformed testing, the average improvement of the combined strategy over testing alone was 3.09% of GDP.

**Table 3:** Model parameter descriptions, primary values, ranges used in sensitivity analyses, and citations to sources used to inform our selections of primary values and ranges.

Parameter	Description	Primary	Range	Sources
$$R_0$$	Basic reproductive ratio	2.5	1–10	^38,66–70^
$$1/\gamma$$	Average infectious period [days]	8	4–12	^71^
$$\rho$$	Infection fatality ratio	0.01	0.005–0.050	^66,72–74^
$$i_0$$	Initial fraction of population infected	0.01	0.001–0.05	–
$$T_{v}$$	Time until vaccine is available [days]	365	183–730	–
*k*	Share of superspreaders	0.1	0.05–0.25	^16,22,39^
$$\alpha$$	Share of infections by superspreaders	0.80	0–1	^16,22,39^
*VSL*	Value per statistical life [10$$^6$$$]	10	1–10	^29,49,55,75–77^
$$\eta$$	Coefficient of relative risk aversion	2.0	1–3	^51,56–58^
*r*	Discount rate [/yr]	0.03	0.015–0.07	^46,54,55^
$$t_R$$	Economic recovery time [yr]	5	0–10	^78,79^
$$\varepsilon _1$$	Type I error rate (1-specificity)	0.03	0.01–0.1	^26,80–83^
$$\varepsilon _{2I}$$	Type II error rate (1-sensitivity) for infecteds	0.25	0.05–0.30	^26,80–83^
$$\varepsilon _{2Z}$$	Type II error rate (1-sensitivity) for superspreaders	0.10	0.02–0.20	^33,84^
$$1/\sigma$$	Average test waiting time [days]	1	0.5–5	–
$$\lambda$$	Self-isolation compliance ratio	0.70	0.1–1	^85–87^
$$1/\delta$$	Average isolation period [days]	14	5–21	–
*p*	Marginal cost of testing at $$\tau \!=\!{1}/{2}$$ [$/test]	50	25–150	^26,63,88–90^
*y*	Marginal cost of distancing at $$x={1}/{2}$$ [$/yr]	70,693	–	^91^
$$\theta$$	Normalized slope of marginal cost curves	0.5	0–1	–

## Discussion

We developed an integrated epidemiological and economic model to compare the performance of two distinct strategies for controlling an infectious disease outbreak: physical distancing and random testing with self-isolation. The model includes a simple representation of superspreading and accounts for false positive and false negative diagnostic testing errors and partial compliance with self-isolation recommendations. The model also incorporates a diminishing marginal value of mortality risk reductions, which is important for generating realistic estimates of willingness-to-pay for large reductions in the risk of death.

The immediate implications of our results pertain to the relative performance of physical distancing versus testing with self-isolation control strategies. We found that for an epidemic similar to the ancestral strain of SARS-CoV-2, an optimized strategy of random testing with voluntary self-isolation can deliver higher net benefits than a physical distancing strategy over a wide range of plausible conditions in our model. The performance of both strategies depend strongly on the transmissibility of the pathogen. There is an intermediate level of transmissibility ($R_0 \approx 2$) at which the net benefits of each strategy is maximized, and as transmissibility increases beyond this level their performance degrades until a higher threshold level of transmissibility is reached beyond which neither strategy can achieve positive net benefits. The performance of both strategies appears to always increase in the lethality of the pathogen, but the performance advantage of the testing policy over the distancing policy is maximized at an infection fatality ratio $\rho \approx 0.015$, which is 1.5 to 3 times higher than the ancestral strain of SARS-CoV-2. We also found an important influence of “superspreading”—whereby a large fraction of transmissions are attributed to a small fraction of infected individuals—on the relative performance of the two policies. Insofar as diagnostic tests are sensitive to viral loads, and individuals with high viral loads are more likely to transmit the pathogen to others, superspreading enhances the relative performance of the testing strategy over the physical distancing strategy.

Our key result is that an optimized random testing strategy can outperform an optimized physical distancing strategy for mitigating COVID-19 or infectious diseases with similar features. One necessary condition for this result to hold is that people comply with the self-isolation guidelines at least 20 percent of the time they receive a positive COVID-19 test (Fig. 4, bottom center graph). This seemingly low threshold level of compliance emerges due to the relatively high overall cost of physical distancing, which affects all individuals on an ongoing basis rather than the smaller number of individuals who test positive at any given time. Our primary value of the self-isolation compliance rate was $\lambda =0.7$, so we assumed reasonably high but not full compliance. Our sensitivity analysis showed that a testing strategy can outperform a distancing strategy as long as $\lambda > 0.2$. This is paired with a high rate of testing ($\tau = 1.0$) and so is similar to full compliance ($\lambda = 1.0$) at a lower rate of testing ($\tau = 0.2$, once every five days). The testing strategy is relatively robust to lower rates of compliance because both the benefits and costs of the testing strategy decline with the rate of compliance: if people fail to isolate after receiving a positive test result, then fewer cases would be prevented *and* lower costs would be imposed. The effectiveness of the testing strategy from a public health perspective would be degraded at lower levels of compliance, but the difference between economic benefits and costs would remain larger than an optimized distancing policy over a reasonably wide range of self-isolation compliance rates. If compliance completely unravels such that $\lambda < 0.2$, then a physical distancing strategy that achieved the optimal distancing fraction ($x = 0.189$) would be more efficient. Naturally, sufficient compliance also would be required for a distancing strategy to perform as predicted. This highlights the crucial role of behavioral reactions to public health policies, which can undermine otherwise well-designed regulations^34–36^.

Another key result is that a combined strategy could not improve on an optimized testing strategy under our primary parameter values with a diminishing VSL. However, we found that a hybrid policy including testing and a modest amount of physical distancing was optimal when we used a constant VSL. More broadly, our Monte Carlo uncertainty analysis using random parameter draws revealed that some combination of distancing and testing outperformed either strategy alone in 27.0% of Monte Carlo iterations, testing alone was optimal in 51.6% of iterations, distancing alone in 17.0%, and no controls in 4.4%. While testing alone outperformed distancing in 59.6% of the iterations, the combined strategies outperformed distancing alone in 78.6% of the iterations. We take these results to suggest that a testing strategy should be given consideration at least on par with a physical distancing strategy for controlling future epidemics. Though not modeled here, another potential advantage of a testing strategy over a distancing strategy is that aggregated testing results could be used to better monitor the state of the epidemic as it evolves, which could allow both regulators and individuals to dynamically adjust their “top-down” control measures and “bottom-up” self-protection behaviors as the epidemic waxes and wanes over time^37^.

A broader implication of our results pertains to the level of performance we should expect from even a well-functioning policy process in the context of a novel epidemic when key parameters cannot be precisely estimated. As Ferguson et al. explained, “Two fundamental strategies are possible: (a) mitigation, which focuses on slowing but not necessarily stopping epidemic spread—reducing peak healthcare demand while protecting those most at risk of severe disease from infection, and (b) suppression, which aims to reverse epidemic growth, reducing case numbers to low levels and maintaining that situation indefinitely. Each policy has major challenges^38^”. The twin peaks in the net benefit curves shown in Figs. 1 and 2 can be associated with these two contrasting strategies. The peaks at the the low levels of $$T_{\tau }$$ in Fig. 2 can be viewed as mitigation strategies, which save a smaller number of lives at a low cost, while the peaks at the high $$T_{\tau }$$ values can be viewed as suppression strategies, which save many more lives at a much higher cost. This distinction also shows up in both panels of Fig. 1. In the top panel, the lower and higher peaks of the dashed line ($$\eta = 0$$) correspond to mitigation and suppression strategies, respectively. In the bottom panel, as the average price of diagnostic tests increases from $25 to $100, the optimal frequency of testing drops rapidly from nearly every day ($$\tau$$ close to 1) down to about once every five days ($$\tau \approx 0.2$$). The former configuration is a suppression strategy while the latter is a mitigation strategy. These results suggest that modest uncertainty about one or more key parameters ($$\eta$$ and *p* among others) can lead to major uncertainty about the optimal policy strategy, to the point where it may be unclear whether the qualitatively distinct strategies of mitigation or suppression should be pursued. When the policy strategy rankings in deterministic models such as the one used here are substantially sensitive to one or more uncertain parameters, it becomes easier to understand the wide range of views about the wisdom of relatively lax versus strict controls among the general public and even many experts. Future studies might shed more light on these issues by employing a richer model of superspreading^39^ and by incorporating endogenous pathogen infectivity to mimic the adaptive evolution of the pathogen over time^40^.

## Methods

We developed a combined epidemiological and economic model that can be optimized by adjusting control variables representing physical distancing and random testing with self-isolation policies. As with similar compartment-based models used in previous studies, the model used here is as parsimonious as possible while including enough detail to address our main research questions^41,42^. Descriptions of all parameters and the primary and low and high values used in our epi-econ model are provided in Table 3.

### Epidemiology

To represent the dynamics of viral spread, illness, and recovery, we couple epidemiological and economic processes using a continuous-time compartment model based on a standard *S-I-R* framework^28,43^. In addition to tracking the fraction of the population who are susceptible (*S*), infected (*I*), recovered (*R*), or dead (*D*), we add a primary compartment for infected individuals with elevated transmissibility (“superspreaders”) (*Z*)^18^. We also added 13 secondary compartments to track the fate of individuals who get tested for COVID-19: nine distinct states of susceptible, infected, superspreading, and recovered individuals who are waiting for a test result ($W_{SS}$, $W_{II}$, $W_{ZZ}$, $W_{RR}$, $W_{SZ}$, $W_{SI}$, $W_{IR}$, $W_{ZR}$, and $W_{SR}$), and susceptible, infected, superspreading, and recovered individuals who are in isolation ($Q_S$, $Q_I$, $Q_Z$, and $Q_R$). While we omit time notation, all upper case variables are dependent upon time, and time derivatives are denoted by over-dot notation. To define the transition rates among these additional compartments, we add parameters to represent the influence of physical distancing on the contact rate, the share of infected individuals who become superspreaders, the frequency of testing, the delay in receiving test results, the false positive and false negative error rates of the tests, and the average testing compliance rate among individuals who receive a positive test result and are asked to self-isolate.

**Figure 6:**
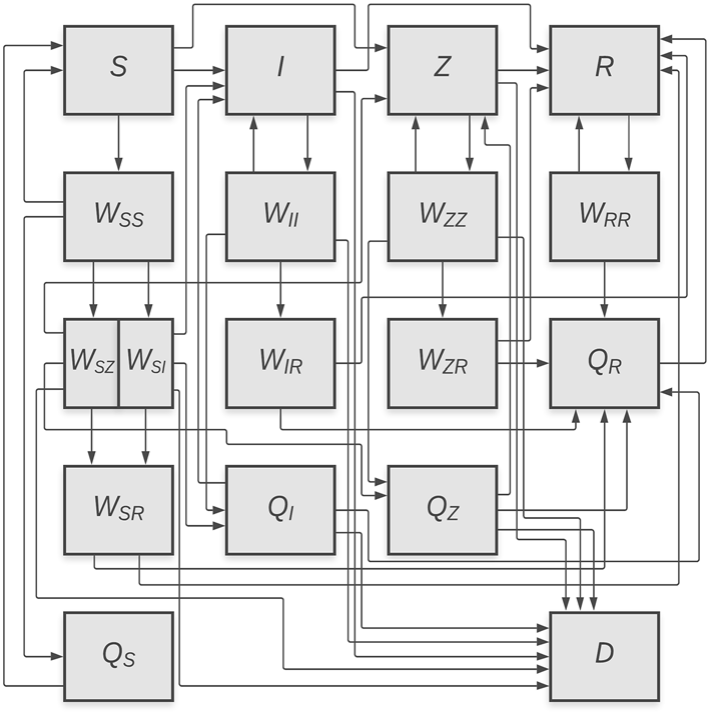
Augmented *S-I-R* compartment model. The *Z* compartment represents superspreaders, *W* compartments represent individuals waiting for test results, and *Q* compartments represent individuals under self-isolation. Lines represent transitions between compartments due to infection, recovery, mortality, and testing.

A flow diagram depicting all routes for the transition of individuals between compartments is shown in Fig. 6, and the model parameters are described in Table 3. The flows indicated by the arrows in Fig. 6 correspond to state transition terms that appear in the equations of motion below. We will describe the elements of several equations of motion in detail; the remaining equations can be interpreted using the same logic.

The equation of motion for susceptible individuals is

1 $$\begin{aligned} \dot{S} = -\left (1-x \right )^2\left[ \beta _I \left (I+ W_{SI} + W_{II}\right )+ \beta _Z \left (Z + W_{SZ} + W_{ZZ}\right )\right] S - \tau S + \sigma \left (1-\varepsilon _1\lambda \right )W_{SS} +\delta Q_S. \end{aligned}$$

The first term on the right hand side of Eq. (1), $(1-x)^2\left[ \cdot \right] S$, represents newly infected individuals. Transmission of the pathogen to a susceptible individual, the total number of which is *S*, can occur by contact with a non-superspreading infected individual, the total number of which is $I+W_{SI}+W_{II}$, or a superspreading infected individual, the total number of which is $Z+W_{SZ}+W_{ZZ}$. $\beta _I$ is the product of the average (baseline, un-controlled) probability of contact between pairs of susceptible and non-superspreading infected individuals and the probability of transmission conditional on contact between them. Likewise, $\beta _Z$ is the product of the average (baseline, uncontrolled) probability of contact between pairs of susceptible and superspreading infected individuals and the probability of transmission conditional on contact between them. The influence of a physical distancing policy is represented by *x*, which is the fractional reduction in the average number of contacts that each individual has with other individuals. The term $(1\!-\!x)$ is squared in Eq. (1) and elsewhere because the number of new infections is proportional to the product of the number who are currently infected and the number who are currently susceptible. If only the infected or only the susceptible individuals remain isolated on a fraction *x* of the occasions when they otherwise would make contact with other individuals, then the rate of new infections would be $(1\!-\!x)\beta S I$. Assuming both types of individuals, infectious and susceptible, remain isolated on a fraction *x* of the occasions when they otherwise would make contact with others, the rate of new infections becomes $(1\!-\!x)^2\beta S I$.

The second term in Eq. (1), $\tau S$, is the number of susceptible individuals who get tested. The third term includes those individuals who were tested as susceptible and receive a true negative test result, $\sigma (1-\varepsilon _1)W_{SS}$, plus those who receive a false positive test result and do not comply with the request to self-isolate, $\sigma \varepsilon _1(1-\lambda )W_{SS}$. The final term, $\delta Q_S$, represents those who were isolated as susceptible that exit isolation.

The equation of motion for non-superspreading infected individuals is

2 $$\begin{aligned} \dot{I}&= \big (1-x\big )^2\big (1-k\big )\big [\beta _I\big (I+W_{SI}+W_{II}\big ) +\beta _Z\big (Z+W_{SZ}+W_{ZZ}\big )\big ]S - \big (\tau +\gamma +m\big )I\nonumber \\ {}&\quad +\sigma \big [(1-\varepsilon _1)+\varepsilon _1\big (1-\lambda \big )\big ]W_{SI} +\sigma \big [ \varepsilon _{2I}+\big (1-\varepsilon _{2I}\big )\big (1-\lambda \big ) \big ]W_{II} + \delta Q_I. \end{aligned}$$

The first term on the right hand side of Eq. (2) includes the $\big (1-k\big )$ share of newly infected individuals who do not become superspreaders. The second term includes those who are tested (at rate $\tau$), those who recover (at rate $\gamma$), and those who die (at rate *m*), where $m= \frac{\rho \gamma }{1-\rho }$ and $\rho$ is the infection fatality ratio^44^. The third term includes those who were tested while susceptible but are currently infected and receive a true negative test result or receive a false positive test result but do not comply with the guidelines to isolate. The fourth term includes those who were tested while infected and are still infected and receive a false negative test result or receive a true positive test result but do not comply. The fifth and final term includes those who are exiting isolation but are still infected.

The equation of motion for superspreading infected individuals is directly analogous to that for non-superspreading infected individuals:

3 $$\begin{aligned} \dot{Z}&= \big (1-x\big )^2k\big [\beta _I\big (I+W_{SI}+W_{II}\big )+\beta _Z\big (Z+W_{SZ}+W_{ZZ}\big )\big ]S - \big (\tau +\gamma +m\big )Z\nonumber \\ &+\sigma \big [(1-\varepsilon _1)+\varepsilon _1\big (1-\lambda \big )\big ]W_{SZ} +\sigma \big [ \varepsilon _{2Z}+\big (1-\varepsilon _{2Z}\big )\big (1-\lambda \big ) \big ]W_{ZZ} + \delta Q_Z. \end{aligned}$$

The first term on the right hand side of Eq. (3) includes the *k* proportion of newly infected individuals who become superspreaders. The second term includes superspreaders who are tested, or recover, or die. The third term includes those who were tested while susceptible but are currently superspreaders and receive a true negative test result or a false positive test result but do not comply with the guidelines to isolate. The fourth term includes those who were tested while superspreaders and are still superspreaders and receive a false negative test result or receive a true positive test result but do not comply. The fifth and final term includes those who are exiting quarantine but are still superspreaders.

The equation of motion for recovered individuals is

4 $$\begin{aligned} \dot{R}&= \gamma \big (I\!+\!Z\big ) + \delta Q_R +\sigma \big (1-\lambda +\lambda \varepsilon _{2I}\big )W_{IR} +\sigma \big (1-\lambda +\lambda \varepsilon _{2Z}\big )W_{ZR}\nonumber \\&\quad + \sigma \big (1-\lambda \varepsilon _1\big )W_{SR} +\sigma \big (1-\lambda \varepsilon _1\big )W_{RR} - \tau R \end{aligned}$$

The first term on the right-hand side of Eq. (4) includes recovered, infected, and superspreading individuals. The second term includes recovered individuals who exit isolation. The third and fourth terms include individuals who were tested while infected or superspreaders but are now recovered and receive a false negative test result or a true positive test result but do not comply with the isolation guidelines. The fifth and sixth terms include individuals who were tested while susceptible then became infected or superspreaders but are now recovered and receive a true negative test result or a false positive test result but do not comply. The seventh and final term includes recovered individuals who are tested.

The remaining equations of motion use elements that are common or analogous to one or more of those described above, so we list them below for completeness but we refrain from explaining each one in turn.

5 $$\begin{aligned} \dot{W}_{SS}&= \tau S - \big (1-x\big )^2\big [\beta _I\big (I+W_{SI}+W_{II}\big )+\beta _Z\big (Z+W_{SZ}+W_{ZZ}\big )\big ]W_{SS} - \sigma W_{SS} \end{aligned}$$

6 $$\begin{aligned} \dot{W}_{II}&= \tau I - \big (\gamma +\sigma +m\big ) W_{II} \end{aligned}$$

7 $$\begin{aligned} \dot{W}_{ZZ}&= \tau Z - \big (\gamma +\sigma +m\big ) W_{ZZ} \end{aligned}$$

8 $$\begin{aligned} \dot{W}_{RR}&= \tau R - \sigma W_{RR} \end{aligned}$$

9 $$\begin{aligned} \dot{W}_{SZ}&= \big (1-x\big )^2k\beta _I\big (I+W_{SZ}+W_{ZZ}\big ) W_{SS}-\big (\gamma +\sigma +m\big ) W_{SZ} \end{aligned}$$

10 $$\begin{aligned} \dot{W}_{SI}&= \big (1-x\big )^2\big (1-k\big )\beta _I\big (I+W_{SI}+W_{II}\big ) W_{SS}-\big (\gamma +\sigma +m\big ) W_{SI} \end{aligned}$$

11 $$\begin{aligned} \dot{W}_{IR}&= \gamma W_{II} - \sigma W_{IR} \end{aligned}$$

12 $$\begin{aligned} \dot{W}_{ZR}&= \gamma W_{ZZ} - \sigma W_{ZR} \end{aligned}$$

13 $$\begin{aligned} \dot{Q}_R&= \gamma \big (Q_I+Q_Z\big ) + \sigma \lambda \big [\varepsilon _1\big (W_{SR}+W_{RR}\big )+\big (1-\varepsilon _{2I}\big )W_{IR} +\big (1-\varepsilon _{2Z}\big )W_{ZR}\big ] - \delta Q_R \end{aligned}$$

14 $$\begin{aligned} \dot{W}_{SR}&= \gamma \big (W_{SI}+W_{SZ}\big ) - \sigma W_{SR} \end{aligned}$$

15 $$\begin{aligned} \dot{Q}_I&= \sigma \lambda \big [\big (1-\varepsilon _{2I}\big )W_{II}+\varepsilon _1 W_{SI}\big ] - \big (\gamma +\delta +m\big )Q_I \end{aligned}$$

16 $$\begin{aligned} \dot{Q}_Z&= \sigma \lambda \big [\big (1-\varepsilon _{2Z}\big )W_{ZZ}+\varepsilon _1 W_{SZ}\big ] - \big (\gamma +\delta +m\big )Q_Z \end{aligned}$$

17 $$\begin{aligned} \dot{Q}_S&= \sigma \varepsilon _1\lambda W_{SS} -\gamma Q_S \end{aligned}$$

18 $$\begin{aligned} \dot{D}&= m\big (I + Z + W_{II} + W_{ZZ} + W_{SI} + W_{SZ} + Q_I + Q_Z\big ) \end{aligned}$$

Equations (1)–(18) comprise the epidemiological model, accounting for superspreaders (*k*), physical distancing (*x*), random testing ($\tau$), the error rates of the tests ($\varepsilon _1$, $\varepsilon _{2I}$, and $\varepsilon _{2Z}$), the duration of self-isolation ($\delta$), and the self-isolation compliance rate ($\lambda$).

To calibrate the transmission coefficients, we used previously published estimates for COVID-19 of the basic reproductive number, $R_0$, the fraction of infected individuals who are superspreaders, *k*, and the fraction of infections attributable to superspreaders, which we denote as $\alpha$. To see how these quantities relate to $\beta _Z$ and $\beta _I$, consider a version of the compartment model that includes superspreaders but excludes physical distancing and testing. The relevant equations of motion are

19 $$\begin{aligned} \dot{S}&= -\left( \beta _Z Z + \beta _I I\right) S \end{aligned}$$

20 $$\begin{aligned} \dot{Z}&= k\left( \beta _Z Z + \beta _I I\right) S - \left( \gamma +m\right) Z \end{aligned}$$

21 $$\begin{aligned} \dot{I}&= \left( 1-k\right) \left( \beta _Z Z + \beta _I I\right) S-\left( \gamma +m\right) I \end{aligned}$$

The number of infections will grow when $\dot{Z}+\dot{I} > 0$, i.e., when

22 $$\begin{aligned} \left( \beta _Z Z + \beta _I I\right) S - \left( \gamma + m\right) \left( Z + I\right) > 0 \end{aligned}$$

The fraction of infected individuals who are superspreaders is $k = Z/(Z+I)$. From this, we can substitute $Z = kI/(1-k)$ into Eq. (22) to get

23 $$\begin{aligned} \left( \tfrac{k}{1-k}\beta _Z+\beta _I\right) IS > \left( \gamma +m\right) \tfrac{1}{1-k}I \end{aligned}$$

Next, substituting $S\!=\!1$ into Eq. (23) and rearranging, we find that when a small number of infected individuals are introduced into a completely susceptible population an outbreak will occur (the number of infected and superspreading individuals will grow) only if

24 $$\begin{aligned} \frac{k\beta _Z+\left( 1-k\right) \beta _I}{\gamma +m} > 1 \end{aligned}$$

The ratio on the left-hand side of expression (24) corresponds to $R_0$ in the superspreader model. Next, note that the fraction of infections caused by superspreaders is

25 $$\begin{aligned} \alpha = \frac{\beta _ZZS}{\left( \beta _ZZ+\beta _II\right) S} \end{aligned}$$

Substituting $Z\!=\!kI/(1-k)$ into Eq. (25) gives a second equation that relates our calibration statistics to the two unknown transmission coefficients,

26 $$\begin{aligned} \alpha = \frac{k\beta _Z}{k\beta _Z+\left( 1-k\right) \beta _I} \end{aligned}$$

Finally, combining Eqs. (24) and (26) and solving for the transmission coefficients gives

27 $$\begin{aligned} \beta _I = \left( \gamma +m\right) \left( \frac{1-\alpha }{1-k}\right) R_0 \end{aligned}$$

and

28 $$\begin{aligned} \beta _Z = \left( \gamma +m\right) \frac{\alpha R_0}{k} \end{aligned}$$

We chose numerical values for most of the epidemiological parameters based on a review of recent empirical studies of COVID-19 transmission in the United States, which are cited in Table 3. The primary values represent our summary judgments of reasonable central estimates for each parameter, and the lower and higher values in the final column of the table, which we use for sensitivity and Monte Carlo analyses, are meant to span most of the plausible range for each parameter.

A few model parameters are incidental and were chosen for practical reasons. In particular, $T_v$, the time until a vaccine is available, is now moot for COVID-19 because effective vaccines have since been widely distributed. So we set $T_v$ to represent a scenario like the early months of the COVID-19 pandemic when vaccines were still on the horizon and physical distancing and testing were two of the main policy levers at that time. We also set $i_0=I_0/N$, the initial fraction of the population infected, at an arbitrarily small value, with no intention to peg the initial period of our model to any specific day early in the pandemic. The particular set of primary parameter values used here is not meant to be definitive, but we do intend them to fall well within the ballpark of realistic estimates for COVID-19, and we intend our sensitivity analyses to cover a wide range of plausible alternative scenarios that could emerge in future waves of COVID-19 or in future unrelated respiratory epidemics.

### Economics

To estimate the economic benefits and costs of epidemiological outcomes simulated by the extended *S-I-R* model, we used four key elements that generalize those in our previous work^45,46^. First, we used an estimate of the “value per statistical life” (VSL) to calibrate a willingness-to-pay function for reduced COVID-19 mortality risks for a representative individual. The VSL is the marginal rate of substitution between income and mortality risk and is the proper accounting value to use in benefit-cost analyses of public health policies that will reduce mortality risks and increase longevity in the general population^47^. However, being a marginal rate of substitution, the VSL is not applicable for valuing non-marginal changes in mortality risks without some adjustment. In our model, mortality risks can change by amounts approaching 0.01 in a single year in our primary scenario, and surpassing that level in some sensitivity analyses, which is several orders of magnitude larger than the risk changes typically considered in benefit-cost analyses of U.S. federal regulations where the VSL is routinely applied. Several recent articles provide a range of perspectives on using the VSL to evaluate COVID-19 policies, including comparisons to other contexts with smaller changes in mortality risks^30,48–50^.

Here we account for the diminishing marginal value of mortality risk reductions by adjusting the VSL for large risk changes. To do so, we use a life-cycle utility model to derive a willingness-to-pay (WTP) function for mortality risk reductions that can be easily calibrated using estimates of the VSL and employed in the coupled model. Our compartment model does not account for age differences, so we develop a WTP function for an ageless representative individual with a flow utility function including a base level of utility $$\kappa$$ and constant relative risk aversion $$\eta$$, constant annual income *Y* [$ yr$$^{-1}$$], constant annual background risk of mortality *b* [yr$$^{-1}$$], and utility discount rate $$\omega$$ [yr$$^{-1}$$]. Similar utility functions including the constant base level of flow utility have been used by previous researchers to study diverse phenomena, from changes in the demand for health care over time to the value of reducing the risks of catastrophic climate change^51–53^. Since we examine epidemic durations up to 2 years, we write the representative individual’s discounted expected utility to explicitly accommodate changes in first and second period mortality rates as follows:

29 $$\begin{aligned} U & =\left( \kappa + \frac{Y_0^{1-\eta }}{1-\eta }\right) +\frac{1-b-m_0}{1+\omega }\left( \kappa +\frac{Y_1^{1-\eta }}{1-\eta }\right) \\ & \quad +\frac{\left( 1-b-m_0\right) \left( 1-b-m_1\right) }{\left( 1+\omega \right) ^2}\sum _{t=2}^{\infty } {\left( \frac{1-b}{1-\omega }\right) ^{t-2}\left( \kappa +\frac{Y_t^{1-\eta }}{1-\eta }\right) }, \end{aligned}$$

where $m_0$ and $m_1$ are the uncontrolled disease fatality risks in the first and second periods. Note that as $\eta \rightarrow 1$, the flow utility function $(Y^{1-\eta })/(1-\eta ) \rightarrow \ln {(Y)}$.

Setting baseline utility (with no controls and no payment) equal to policy utility (with controls imposed and *WTP* subtracted from income in the first period only) and then solving for *WTP* gives the following explicit expression for willingness-to-pay:

30 $$\begin{aligned} \textit{WTP}\left( \Delta m_0,\Delta m_1\right) = Y-Y\left[ 1-\left( 1-\eta \right) A\frac{\textit{VSL}}{Y}\right] ^{\frac{1}{1-\eta }}, \end{aligned}$$

where

31 $$\begin{aligned} A = \left( b+\omega \right) \left[ \frac{\Delta m_0\left( 1+\omega -m_1\right) +\Delta m_1\left( 1-b-m_0\right) +\Delta m_0\Delta m_1}{\left( b+\omega \right) \left( 1+\omega \right) }\right] , \end{aligned}$$

and where the VSL is defined as the marginal rate of substitution between mortality risk and first-period income,

32 $$\begin{aligned} \textit{VSL} \equiv -\frac{\partial U/\partial m_0}{\partial U/\partial Y_0} = \frac{Y_0^{\eta }}{b+\omega }\left( \kappa +\frac{Y^{1-\eta }}{1-\eta }\right) . \end{aligned}$$

We used a background mortality rate equal to the inverse of expected lifespan at birth in the U.S., $$b=1/77$$ per year. For the VSL, we used a primary value of $10 million, which is consistent with U.S. federal government recommendations, and in a sensitivity analysis we examined values as low as $1 million^54,55^. For $$\eta$$, we used a primary value of 1, and in sensitivity analyses we examined values from 0 to 3^51,56–58^. Applying a constant VSL of $10 million (assuming $$\eta = 0$$) to a 1-year mortality risk reduction of 0.01 gives a predicted WTP of $100,000, which is nearly 1.5 times average annual income in the United States. Using $$\eta > 0$$ constrains WTP to be no larger than annual income, and for a representative individual with annual income *Y* = $70,000, at $$\eta =$$ 1 and 3 the predicted WTP is 0.76 and 0.49 times annual income.

We linked the opportunity cost per day of physical distancing and self-isolation to average daily earnings, similar to several previous optimal control studies of COVID-19 policies^59–61^. To specify the cost function, we assumed that the marginal costs of distancing increases linearly with the fractional reduction of labor supplied to the workforce. The overall reduction in labor supply depends on both the distancing fraction, *x*, and the testing rate, $\tau$, where the effect of the latter is determined by the number of individuals who are in isolation. The fractional reduction of labor supply on day *t* is

33 $$\begin{aligned} x'_t = \frac{1}{N}\left[ x\left( S_t+I_t+\cdots \right) +\left( Q_{St}+Q_{It}+\cdots \right) \right] . \end{aligned}$$

We use *y* to denote average earnings per day prior to the outbreak, and we use $\theta$ to denote the variation in marginal costs over the full range of $x'_t$ from 0 to 1. The marginal cost of the first unit of withdrawn labor (when $x'_t=0$) is $y-\theta y/2$ and the marginal cost of the last unit (when $x'_t=1$) is $y+\theta y/2$. Therefore, the average cost per unit of withdrawn labor on day *t* for an arbitrary fractional reduction in labor supply $x'_t$ is

34 $$\begin{aligned} \frac{1}{x'_t}\int _0^{x'_t}{\left( y-\tfrac{\theta }{2}y+\theta yz\right) dz} = y\left[ 1-\left( 1-x'_t\right) \tfrac{\theta }{2}\right] . \end{aligned}$$

For $\theta$, we used 0.5 as our primary value, and in a sensitivity analysis we examined a range from 0, which implies constant marginal costs, to 1, which means the marginal cost of the first and last units of withdrawn labor are 0.5*y* and 1.5*y*. If physical distancing can be accomplished by some people working remotely, traveling less, minimizing face-to-face meetings, and so on, without being furloughed or losing their jobs for the duration of the lockdown period, then $\theta$ would be positive. Considering that some fraction of the workforce can physically distance at moderate levels with relatively little loss of productivity, we view $\theta =0.5$ as a middle-of-the-road primary value.

Given the above cost function specification, on day *t* the value of lost output due to physical distancing is

35 $$\begin{aligned} \ell _t = x\left( S_t+I_t+\cdots \right) \left[ 1-\left( 1-x'_t\right) \tfrac{\theta }{2}\right] y \end{aligned}$$

and due to isolation after testing is

36 $$\begin{aligned} q_t = \left( Q_{St}+Q_{It}+\cdots \right) \left[ 1-\left( 1-x'_t\right) \tfrac{\theta }{2}\right] , \end{aligned}$$

where the suppressed state variables in Eq. (35) include all compartments subject to distancing, and the suppressed state variables in Eq. (36) include all compartments representing individuals who are currently in isolation after receiving a positive test result.

Using analogous assumptions, we specified the cost of administering diagnostic tests on day *t* as

37 $$\begin{aligned} c_t = \tau \left( S_t+I_t+Z_t+R_t\right) \left[ 1-\left( 1-\tau \right) \tfrac{\theta }{2}\right] p, \end{aligned}$$

where *p* is the marginal cost of testing when $$\tau =0.5$$. Our primary estimate of $$p=$$ is $50, which is a generous price for rapid antigen tests. Paul Romer has suggested that the true marginal cost of rapid antigen tests is around $10, and high nominal prices are due to a monopoly markup^62^. In a sensitivity analysis we examined a range from $25 to $150, the upper end of which is a common price for PCR tests^26,63^.

Finally, we assumed that the economy will not immediately recover from the additional shock caused by the disruption to labor supply from physical distancing and isolation and the resources diverted to support a large-scale diagnostic testing program. We represent the output gap induced by the controls as the time averaged per capita output lost during the period of controls,

38 $$\begin{aligned} y'_H - y_H = \frac{1}{N}\left[ \frac{1}{T_x}\sum _{t=0}^{T_x}{\ell _t} +\frac{1}{T_{\tau }}\sum _{t=0}^{T_{\tau }}{\left( q_t+c_t\right) }\right] , \end{aligned}$$

where $y'_H$ is the counterfactual per capita economic output on day $H=\max \left( T_x,T_{\tau }\right)$, when the controls are ended (i.e., per capita output on day *H* along the original growth path), and $y_H$ is actual (reduced) per capita output on day *H*. We assumed that the output gap will close at an exponential rate, $\varphi$. To specify this rate, we assumed that closing the gap by 95% will take $t_R$ years, so $\varphi = -\frac{1}{t_R}\ln {(0.05)}$. In our primary scenario $t_R = 5$ years, and in a sensitivity analysis we examined recovery times between 0 and 10 years.

### Policy comparisons

We used the model to compare an optimized physical distancing policy to an optimized random testing and self-isolation policy, and to investigate whether a combined policy can perform better than either one alone. The objective of each policy is to maximize net benefits, which is the difference between the value of lives saved and the sum of lost economic output and the cost of testing:

39 $$\begin{aligned} \textit{NB} = \underbrace{\textit{WTP}\left( \Delta m_0,\Delta m_1\right) }_{{\small {\begin{array}{c} {\text {Value of reduced}} \\ {\text {mortality risks}} \end{array}}}} - \underbrace{\sum _{t=0}^{T_x}{\ell _t e^{-rt}}}_{{\small {\begin{array}{c} {\text {Output lost due}} \\ {\text {to distancing}} \end{array}}}} - \underbrace{\sum _{t=0}^{T_{\tau }} {\left( q_t+c_t\right) e^{-rt}}}_{{\small {\begin{array}{c} {\text {Output lost due to}} \\ {\text {isolation }} \& {\text{ testing}} \end{array}}}} - \underbrace{ N\left( y'_H-y_H\right) \sum _{t=H}^{\infty }{e^{-(r+\varphi )t}} }_{{\small {\begin{array}{c} {\text {Output lost}} \\ {\text {during recovery}} \end{array}}}}, \end{aligned}$$

where *WTP*$\left( \Delta m_0,\Delta m_1\right)$ represents the monetized value of the reduced mortality risks computed as described above, and *r* and $\varphi$ are the discount rate and the economic recovery rate [day$^{-1}$].

The assumption that vaccine arrival in period $T_v$ will halt the epidemic is an important modeling choice. It is clearly unrealistic with respect to the current COVID-19 pandemic, yet we maintain this assumption as a convenient means of focusing on what may be the only feasible mitigation measures early in an emerging epidemic when vaccines or effective treatments are unlikely to be available for some time. In future work, it would be useful to conduct an expanded analysis including additional control measures such as vaccines and treatments to gain a better understanding of how these might be best deployed in place of or alongside the control measures we examine here.

To streamline the policy scenarios while maintaining comparability among them, we considered policies with a fixed distancing fraction (*x*) imposed for a limited number of days ($T_x$), policies with a fixed testing rate ($\tau$) implemented for a limited number of days ($T_{\tau }$), or policies that combine the two control strategies. Therefore, we solved optimization problems with at most four control variables: $[x,T_x,\tau ,T_{\tau }]$. To minimize the risk of mistaking a local for a global optimum, for the physical distancing policy we computed net benefits for all combinations of $x \in [0.025,0.05,...,1]$ and $T_x \in [5,10,...,T_{v}]$, and for the testing policy we computed net benefits for all combinations of $\tau \in [0.025,0.05,...,1]$ and $T_{\tau } \in [5,10,...,T_{v}]$. To find the optimal (or a nearly optimal) combined policy, we first conducted a coarse grid search over the entire control space where the grid was constructed from 7 evenly spaced values along the full ranges of the four control variables. Then, from the best starting value among those $7^4 = 2,401$ combinations, we used a stochastic gradient search algorithm to improve the solution and reduce the risk that we remained trapped in a local but not global maximum of the net benefit function.

An alternative treatment of the optimization problem could allow continuous adjustment of *x* and $\tau$ over the course of the epidemic, in which case we could apply a dynamic optimization approach similar to that in our previous work^46^. The approach we used here is easier to work with and is arguably as or more realistic. Setting *x* or $\tau$ to a precisely desired level for a precisely targeted length of time is an idealization, but we can imagine a public policy that would attempt to implement such a program. Precisely adjusting *x* or $\tau$ on a continuous basis in response to the day-to-day evolution of the epidemic seems even less realistic. The quantitative details would surely differ, but we would expect to find a broadly similar overall pattern of comparative performance of the control strategies in a continuously optimized model.

The objective function in Eq. (39) is defined by comparison to a scenario with no controls, either “top-down” controls by the government or “bottom-up” controls due to voluntary distancing or other self-protective measures. A completely uncontrolled scenario seems unlikely because we would expect people to modify their behaviors in response to elevated health risks even in the absence of top-down policy interventions^64,65^. Nevertheless, we expect the net benefits of each policy relative to an uncontrolled scenario to accurately rank the economic efficiency of the policies as long as any crowding-in or crowding-out of self protective behaviors is neutral with respect to the controls, *x* and $\tau$.

## Data Availability

Replication code that supports the findings of this study can be found at https://bitbucket.org/stevenewbold/covid-testing-public/.
